# High Levels of Admixture in *Anopheles gambiae* Populations from Côte d’Ivoire Revealed by Multilocus Genotyping

**DOI:** 10.3390/insects13121090

**Published:** 2022-11-26

**Authors:** Naminata Tondossama, Zanakoungo I. Coulibaly, Issouf Traoré, Bérenger A. Ako, Danielle D. Zoh, Chiara Virgillito, Négnorogo Guindo-Coulibaly, Paola Serini, Fabrice K. Assouho, Ibrahima Dia, Andre O. Touré, Maurice A. Adja, Beniamino Caputo, Alessandra della Torre, Verena Pichler

**Affiliations:** 1 Laboratoire de Biologie et Santé, UFR Biosciences, Université Félix Houphouet Boigny Cocody, Abidjan 01 BP V34, Côte d’Ivoire; 2 Entomology and Herpetology Unit, Institut Pasteur de Côte d’Ivoire, Abidjan 01 PB 490, Côte d’Ivoire; 3 Malaria Unit, Institut Pasteur de Côte d’Ivoire, Abidjan 01 PB 490, Côte d’Ivoire; 4 Institut Pierre Richet/Institut National de Santé Publique, Bouaké 01 BP 1500, Côte d’Ivoire; 5 Dipartimento di Sanità Pubblica e Malattie Infettive, Istituto Pasteur Italia-Fondazione Cenci-Bolognetti, Università di Roma “La Sapienza,” Piazzale Aldo Moro, 5, 00185 Rome, Italy; 6 Pôle de Zoologie Médicale, Institut Pasteur de Dakar, 36 Avenue Pasteur, Dakar BP 220, Senegal

**Keywords:** mosquito, malaria vectors, *Anopheles gambiae*, *Anopheles coluzzii*, diagnostics, genomic admixture, Côte d’Ivoire, Africa

## Abstract

**Simple Summary:**

*Anopheles gambiae* and *An. coluzzii* are two mosquito species with the most prominent role in transmitting malaria parasites to humans in the Afrotropical region. They are morphologically indistinguishable, but their ecological and behavioral differences affect their geographical distribution and may impact their role as malaria vectors and their response to malaria control interventions. A few genomic markers differentiate the two species and allow them to be consistently identified across most of their range. We here report the presence of two populations in Côte d’Ivoire characterized by an admixed pattern of these markers and try to understand their nature. Results do not support the hypothesis that the observed patterns are due to the current crossing between the two species, highlighting the constraints of currently available markers in clarifying the origin of the “unusual” populations in the country. Further analysis exploiting a larger set of markers will eventually solve this puzzle and allow a better understanding of its potential impact on malaria transmission and control.

**Abstract:**

*Anopheles coluzzii* and *An. gambiae*—the two most recently radiated species of the *An. gambiae* complex and the major Afrotropical malaria vector species—are identified by markers in the X-centromeric IGS rDNA region. Putative IGS-hybrids are rarely found in the field, except in restricted areas where genomic studies have led to the hypothesis that the observed IGS-patterns are due to cryptic taxa rather than to hybridization between the two species. We investigated the genome-wide levels of admixture in two villages in Côte d’Ivoire where high levels of IGS-hybrids have been detected, confirming unparalleled high frequencies in the coastal village. Genotyping of 24 Ancestry Informative Markers (AIMs) along the three chromosomes produced discordant results between the IGS-marker and the multilocus genotype obtained for AIMs across the whole genome (29%) as well as AIMs on chromosome-X (considered to be fundamental for species reproductive isolation) only (21%). Results highlight a complicated pattern of admixture that deserves deeper genomic analyses to understand better possible underlying causes (from extensive processes of hybridization to the existence of different cryptic taxa), and stress the need of developing advanced diagnostics for *An. coluzzii, An. gambiae* and putative new taxa, instrumental for assessing taxon-specific epidemiological characters.

## 1. Introduction

The *Anopheles gambiae* complex comprises some of the most important malaria vectors present in sub-Saharan Africa. The nine recognized species in the complex are morphologically indistinguishable, but exhibit important differences at the ecological, behavioral, and epidemiological level, including their ability to vector efficiently the *Plasmodium* parasite [1]. While initially only crossing experiments could highlight the existence of such cryptic species, during the past few decades the increasing availability of cytogenetic and molecular techniques has allowed the detection of important sub-structuring within some of the species of the complex, leading eventually to the description of new taxonomic units. This has led to the split of the species *An. quadriannulatus* into *An. quadriannulatus* and *An. amharicus* [2], *An. bwambae* into *An. Bwambae,* and *An. fontenillei* [3], as well as *An. gambiae* s.s. into *An. gambiae* (formerly defined as molecular form S) and *An. coluzzii* (formerly defined as molecular form M) [2,4]. The latter two species represent the most efficient malaria vectors within the complex, owing to their strong anthropophilic and anthropophagic tendencies.

The two species are known to freely mate and produce viable progeny under laboratory conditions but premating and post-mating mechanisms contribute to their reproductive isolation in the field [5]. However, residual hybridization between the two species has very relevant implications. First, it has affected the effectiveness of insecticide-based vector control interventions by allowing the transfer from one species to the other of adaptive mutations, the most well-studied being the knock-down-resistance (kdr) mutation L1014F (or L995F; [6,7,8]) within the *vgsc* gene, highly associated with resistance to pyrethroids. Second, knowledge on ongoing hybridization is relevant for the correct planning of malaria vector control strategies based on releases of genetically manipulated males [9,10].

*Anopheles gambiae* and *An. coluzzii* are defined based on species-specific SNPs within the IGS region of the X-centromeric rDNA (IGS-marker; [4,11]) and the discrimination of the two species is commonly performed by molecular methods able to identify such polymorphisms [12,13,14]. Additionally, the *An. coluzzii*-exclusive insertion of a SINE situated in the X-centromere close to the rDNA region (SINE200 X6.1, hereafter SINE-marker) is frequently used to identify the two species [15]. Individuals characterized by heterozygous patterns are considered as putative hybrids and their sporadic finding in the field (on average <0.2%; [5]) represented the main proof of reproductive isolation based on which they have been raised to different taxa. Nowadays, all evidence of ecological interspecific differentiation—from larval site preferences [16,17,18] to capacity of dispersal by wind [19,20]—are based on specimens’ identification by the above markers.

In the last years, the *Anopheles gambiae* 1000 Genome (Ag1000G) consortium (https://www.malariagen.net/mosquito/ag1000g accessed on 24 July 2022) has provided strong evidence that IGS-identified specimens are consistent with a panel of 506 SNPs across the whole species’ genomes that are virtually fixed between them in most sub-Saharan Africa and are thus defined as Ancestry Informative Markers (AIMs) [8,21]. Only coastal populations at the western extremes of the two species’ range were shown to be characterized by stable frequencies >20% of putative hybrids [22,23,24,25,26,27] as identified by the IGS-marker (hereafter, IGS-hybrids), leading to the definition of this geographical region as High Hybridization zone [28]. Further investigations on populations from Guinea Bissau allowed to describe patterns of apparent admixture across the whole genome, leading to hypothesize the existence of a further cryptic form carrying an *An. gambiae*-like X-chromosome and *An. coluzzii*-like autosomes [25]. Whole Genome Sequence (WGS) data from the Ag1000G consortium provide support to the admixed nature of these populations and attributed to them an ”uncertain species status” due to the presence of a mixture of species-specific AIMs across the genome [8,9].

At the local level, high frequencies of IGS-hybrids in larval samples from Burkina Faso led to the hypothesis of the existence of further cryptic taxa, named GOUNDRY-form [29,30]. This was later shown to represent an admixed population descended from both *An. coluzzii* and an additional cryptic taxon named *Anopheles* TENGRELA [31]. On the other hand, high frequencies of IGS-hybrids in Mali were shown to represent temporary break-downs of reproductive isolation resulting in introgressive hybridization of adaptive alleles conferring resistance to pyrethroid insecticides (e.g., kdr alleles) [6,32].

Overall, these pieces of evidence suggest that high frequencies of IGS-hybrids are indicative of relevant biological phenomena—from novel forms/taxa to introgressive hybridization—which could impact the success of malaria vector control strategies. For this reason, we are here following up the report of the presence of IGS-hybrids at frequencies >20% and ~5% in a coastal and in an inland village in Côte d’Ivoire, respectively [33]. The presence of these IGS-hybrids has been confirmed in different seasons of the year at frequencies varying from 21% to 33% in the coastal village, suggesting this may be a stable phenomenon and not only a temporary breakdown of reproductive isolation. Moreover, PCR-genotyping of two AIMs on chromosome-3 showed occurrence of autosomal introgression [33]. Herein, we present the results of genotyping of a panel of AIMs across the genome in specimens sampled during the same time period and in the same two villages.

## 2. Materials and Methods

The study was carried out in two villages of Côte d’Ivoire, both of which are sentinel sites of the National Malaria Control Program (NMCP): Ayame (5.60216° N and 3.09290° W) a coastal village in the southeast region, and Pétessou (7.55505° N and 5.06052° W), situated in the central region. Frequencies of 27% and 5% of *An. coluzzii/An. gambiae* hybrid IGS-genotypes were reported in Ayame and Pétessou, respectively [33].

Mosquitoes were collected during December 2018 and in March, May, and October 2019 by pyrethrum spray collections for four consecutive days/month in five randomly selected houses/village. The protocol for this study was reviewed and approved by the National Research Ethics Committee of Côte d’Ivoire (023-18/1VISHP/CNER-kp). Free and informed consent was obtained from the heads of households for collection in their rooms. The collected mosquitoes were identified as *An. gambiae* s.l using the key of Gillies and Coetzee [34] and kept dry in microtubes containing silica gel.

DNA of *An. gambiae* s.l. females was extracted from single legs using DNAzol (MRC. Inc., Cincinnati, OH, USA) following the protocol described by Rider et al. [35] and extracted DNA was stored at −20 °C for future analysis. All collected specimens were genotyped for species-specific SNPs in the IGS-rDNA region by the IMP-PCR approach described by Wilkins et al. [14]. A multilocus approach [28] was applied to genotype a panel of 24 Ancestry Informative Markers (AIMs) [8] in randomly selected specimens from Ayame and Pétessou. To this aim, DNA was extracted from whole body using the DNA Blood and Tissue Kit (QIAGEN, Hilden, Germany) following the manufacturer protocol. 

Subsequent library preparation as well as sequencing and filtering of sequences was performed at the Polo d’Innovazione di Genomica, Genetica e Biologia Srl within the framework of the Infravec2 project. Libraries for the amplicons spanning the 24 AIMs (10 on chromosome-X centromere, 6 on chromosome-2L centromere, 2 on chromosome-2R, 4 on chromosome-3R telomere and 2 on chromosome-3L centromere; Supplementary Material: Table S1; [28]) were prepared in accordance with RhAmpseq Library preparation kit (©2019 Integrated DNA Technologies Inc., Coralville, IA, USA) using the primer sequences listed in Table S1, and sequencing was performed using the NextSeq platform (2 × 150 paired end). The subsequent bioinformatic analysis was performed following manufacturers guidelines developed to analyze the rhAmpSeq sequencing data. Each sequenced sample was trimmed for adapter sequences during demultiplexing with bcl2fastq v2.20.0.422 software (2019 Illumina, Inc. San Diego, CA, USA) aiming at a preliminary assessment on the overall quality of the produced sequences. Quality control was done using FastQC tool [36]. The reads were aligned to the AgamP4.12 reference genome using the program BWA v0.7.15- r1140 (Burrows-Wheeler Aligner tool, [37]). A sorted-by-position aligned BAM file was produced for each of the 88 samples. Recalibration of base quality scores was performed using two GATK4 v4.2.5.0 packages: the BaseRecalibrator and the ApplyBQRS in order to identify and correct possible systematic errors that arose during the sequencing step when calculating the base quality score. The function TrimPrimers of the fgbio v0.5.1 program was used to trim the primer sequences from the amplicons to avoid any contribution of these sequences to the wild-type variation. The GATK4 packages HaplotypeCaller, CombineGVCFs and GenotypeGVCFs [38] were used to detect the variants for the 24 AIMs described by Caputo et al. [28]. The variants were filtered based on quality by means of the GATK package VariantFiltration [38].

## 3. Results

A total of 277 specimens were successfully identified by IGS-PCR (106 IGS-*An. coluzzii*, 109 IGS-*An. gambiae* and 62 IGS-hybrids). IGS-hybrids were found at frequencies of 38% and 7% in the coastal and inland village, respectively (Table 1).

**Figure 1:**
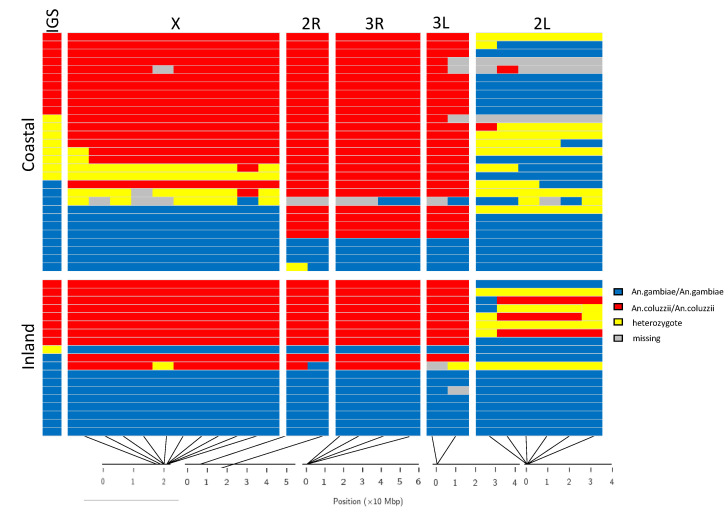
Results of multilocus genotyping of IGS-*An. gambiae,* IGS-*An. coluzzii* and IGS-hybrid specimens from the coastal (Ayame) and the inland (Petessou) villages in Côte d’Ivoire. Each row represents an individual mosquito and columns represent genotyped Ancestry Informative Markers [21]. The first column represents the species ID based on the IGS diagnostic marker. Below the figure the approximate position of AIMs on chromosomal arms. Blue = homozygote for *An. gambiae* specific alleles, red = homozygote for *An. coluzzii* specific alleles, yellow = heterozygote, grey = NA.

Eighty-eight specimens (61 and 27 from the coastal and inland village, respectively) were processed by amplicon sequencing. Genotyping was not successful for 40 specimens, likely due to low quality/quantity of available DNA. In the 48 successfully genotyped specimens (coastal: 10 IGS-*An. coluzzii*, 11 IGS-*An. gambiae*, 8 IGS-hybrids; inland: 8 IGS-*An. coluzzii*, 10 IGS-*An. gambiae*, 1 IGS-hybrid) the genotyping success was >90% for each of the 24 AIMs analyzed and thus none of them was excluded from the analysis (Figure 1, Supplementary Material: Table S2).

Following suggestions by Caputo et al. [28], we defined a minimum number of consensus between species-specific variants to define pure *An. gambiae*, *An. coluzzii*, F1 and admixed specimens. No discordant AIMs were allowed for loci on chromosome-X, while a flexibility of 1 discordant species-specific variant was allowed for the 8 autosomal loci on chromosome arms 2R, 3R and 3L, in order to account for low levels of intraspecific autosomal polymorphism. Loci on chromosomal arm 2L were not considered to define species due to the widespread introgression from *An. gambiae* into *An. coluzzii* of the 2L centromeric region where the *vgsc* gene—carrying possibly *kdr* mutations involved in insecticide resistance—is located.

In both villages, *An. coluzzii* specimens showed consistent IGS and mulitlocus genotypes (N = 18; Table 2). In the inland village, 2 out of 8 IGS-*An. gambiae* specimens and the only IGS-hybrid genotyped were discordantly identified by the two genotyping approaches: one IGS-*An. gambiae* was genotyped as *An. coluzzii* by the mulitlocus approach, one IGS-*An. gambiae* showed a highly admixed genome and the only IGS-hybrid was genotyped as *An. gambiae* by all species-specific AIMs. In the coastal village, 7 out of 11 IGS-*An. gambiae* successfully genotyped were identified discordantly: one was identified by all AIMs as *An. coluzzii*, while 6 were defined as admixed by the multilocus approach. Interestingly, of these 6 specimens, two showed signs of admixture on both autosomes and X-chromosome, while 4 carried an *An. gambiae*-like X-centromere and all *An. coluzzii* specific autosomal markers (with exception of markers on chromosomal arm 2L). Of the eight specimens identified as IGS-hybrids, four were genotyped as pure *An. coluzzii* by the multilocus approach, while the remaining four carried signs of admixture on chromosome-X and predominantly *An. coluzzii*-like autosomal AIMs.

Overall, 71% (34/48) of specimens identified based on IGS markers (100% of *An. coluzzii*; 57% of *An*. *gambiae* and 44% of IGS-hybrids) show consistent AIM-genotypes. On the other hand, IGS-*An. gambiae* are characterized by either admixed (33%, i.e., 7/21) or *An. coluzzii*-like (10%, i.e., 2/21) AIMs, while IGS-hybrids are characterized by either *An. coluzzii*-like (44%, i.e., 4/9) or *An. gambiae*-like AIMs (11%, i.e.,1/9).

Observing the data based on results of the multilocus genotype, the frequency of specimens identified as *An. gambiae* decreases from 44% (IGS; i.e., 21/48) to 27% (AIMs; i.e., 13/48), while the percentage of specimens identified as either *An. coluzzii* or admixed increases from 37% (18/48) to 50% (24/48) and from 19% (9/48) to 23% (11/48), respectively. The 11 specimens with admixed multilocus genotype are characterized by either admixed (64%) or *An. gambiae*-like (36%) chromosome-X AIMs, and in most cases by *An. coluzzii*-like autosomal AIMs.

Focusing on the X-centromeric region (considered instrumental for the two species reproductive isolation; [39]), 21% discordances are observed between IGS-genotypes and the genotype resulting from the 10 AIMs in the region: 5 IGS-hybrids characterized by either *An. coluzzii* AIMs (N = 4) or *An. gambiae* AIMs (N = 1) and 5 IGS-*An. gambiae* characterized by either admixed AIMs (N = 3) or *An. coluzzii* ones (N = 2). Interestingly both the IGS and the multilocus approach identify signature of admixture in the X-centromere in a similar number of specimens (i.e., 9 IGS-hybrids vs 7 specimens with admixed X-centromeres AIMs), but these are not the same individuals.

In 21% of specimens, chromosome-X AIM-genotypes and autosomal AIMs on chromosomal arms 2R, 3R, and 3L produce inconsistent results. These are due to (i) 4 individuals characterized by *An. Gambiae* AIMs on chromosome-X (and *An. Gambiae* IGS-genotype) and *An. Coluzzii* AIMs on autosomes; (ii) 6 individuals characterized by admixed AIMs on chromosome-X and *An. Coluzzii* (N = 5) or *An. Gambiae* (N = 1) autosomal AIMs.

Results of the genotyping of AIMs on chromosomal arms 2L show strong signals of introgression in all individuals characterized by *An. Coluzzii* IGS and AIMs, as well as in those showing hybrid/admixed genotypes.

## 4. Discussion

Herein, we report IGS-hybrids in Côte d’Ivoire at frequencies rarely observed across the species range, as well as incongruences between IGS-diagnostic markers and chromosome-X AIMs and between chromosome-X and autosomal AIMs.

The high IGS-hybrid frequency observed in the coastal village (38%) are unparalleled even by frequencies in the putative high hybridization zone at the westernmost extremes of the species range (<25%; [22,23,24,25,26,27]), but consistent with those resulting from CDC collections carried out in the same village in 2018–2019 (21–33%; [33]. Notably, the literature data describing species composition in Côte d’Ivoire (Supplementary Material: Table S3) either do no report presence of IGS-hybrids, or report them at frequencies ranging from <0.5% [40,41] to 11% [42]. Several explanations may account for this variability in the observed frequency of IGS-hybrids.

First, Ayame village may lay within a region of high hybridization triggered by balanced frequencies of *An. coluzzii* and *An. gambiae* [33], which may favor high levels of hybridization due to increased inter-specific contact, as hypothesized by Pombi et al. [5]. This may support the hypothesis of a temporary breakdown of reproductive barriers restricted to Cote d’Ivoire south-east region in 2018–2019, leading to bouts of hybridization and gene-flow and, thus, important variations in frequency of IGS-hybrids within close years [32]. Identification of further *An. gambiae* samples from different years could help to confirm or dispute this hypothesis.

However, differences between frequencies reported herein and the literature data could also be due to methodological biases which can lead to an underreporting of putative hybrids in scientific publications. First, reporting of a few IGS-hybrids may be considered a neglectable information (or even out of scope) in the case of papers focusing on investigating epidemiologically relevant factors—such as insecticide resistance or other aspects of *An. coluzzii* and *An. gambiae* bionomics directly linked to malaria transmission. Second, the choice of the diagnostic marker used to identify the two species can impact observed frequencies of putative hybrids. In fact, except for Caputo et al. [33], all recent papers reporting the presence of putative hybrids in Cote d’Ivoire (Supplementary Material: Table S3) use the PCR-amplification of an *An. coluzzii*-specific X-centromeric SINE insertion as identification method [15]. Although this method provides results mostly consistently with IGS-PCR, it has been highlighted that—being based on a single copy and irreversible SINE200 insertion—it is not subjected to peculiar evolutionary patterns which instead affect rDNA repeats carrying the IGS-marker, thus leading to a lower rate of heterozygote/hybrid patters compared to the IGS-PCR [43]. In fact, IGS-based identifications can overestimate frequencies of putative hybrids compared to SINE-based identifications, due to an incomplete homogenization of the rDNA arrays leading to the co-existence of *An. coluzzii* and *An. gambiae* specific IGS-sequences on single chromosomes, as shown in hemizygous males [44].

Results of genotyping of 18 AIMs on chromosomal arms X, 2R, 3R, and 3L allow to dispute the hypothesis of a current breakdown of reproductive barriers between *An. coluzzii* and *An. gambiae* since no individuals characterized by consistent heterozygous IGS and AIM patterns (expected for F1 hybrids) are observed. The presence instead of IGS-hybrids carrying concordant homozygous AIM patterns across the genome does support the hypothesis of the presence of admixed IGS arrays which have not been homogenized yet by the process of concerted evolution.

Discordances between the AIMs situated on chromosome-X and on chromosomal arms 2R, 3R, and 3L further complicate the picture. Patterns of admixture seem to be different from what is observed in coastal Guinea Bissau and The Gambia, where linkage among autosomal AIMs is apparently lower than in Côte d’Ivoire [28]. Anyway, it needs to be acknowledged that the sample size analyzed herein is low and genotyping of a higher number of specimens will be needed to provide a more reliable picture and to compare the observed patterns of admixture with that observed in the so-called high hybridization zone in far-West Africa.

From the methodological perspective, the consistency of results of the multilocus genotyping performed herein with those of the PCR-genotyping of only two AIMs on chromosome-3 (i.e., 3R:42848 and 3L:129051) reinforces results obtained by Caputo et al. [33], as well as the value of the two chromosome-3 AIMs in revealing signatures of genomic admixture when associated to IGS-diagnostics. However, it should be noted that the multilocus approach allows the genotyping of 10 AIMs in the chromosome-X centromeric region where also the IGS-marker is located. This allows a direct comparison between these markers and, possibly, a more reliable representation of this so called “speciation island”, where genes implicated in reproductive isolation between *An. coluzzii* and *An. gambiae* are believed to be located [39].

## 5. Conclusions

Overall, the present results highlight the growing problem of correctly interpreting high frequencies of heterozygous IGS-genotypes, conventionally defined as putative hybrids between *An. coluzzii* and *An. gambiae* and generally reported at frequencies <0.2% [5]. As in the cases of populations at the westernmost extreme of the species range [22,23,24,25,27,28] and of the Tengrela population in Burkina Faso [31], the finding of unparalleled frequencies of IGS-hybrids in the coastal village of Côte d’Ivoire (reported also by [33]) reveals a complicated pattern of admixture that deserves deeper genomic analyses to allow a better understanding of possible underlying causes, from extensive processes of hybridization to the existence of further cryptic taxa.

## Figures and Tables

**Table 1:** Frequency of IGS-*An. coluzzii,* IGS-*An. gambiae* and IGS-hybrids (GA/CO) in a coastal (Ayame) and in an inland (Petessou) village in Cote d’Ivoire. N = number of specimens identified by IGS markers [14].

Sampling Site	N	*An.coluzzii*	GA/CO	*An.gambiae*
Coastal	139	0.34	0.38	0.28
Inland	138	0.43	0.07	0.51

**Table 2:** Results of multilocus genotyping of IGS-*An. Gambiae,* IGS-*An. Coluzzii,* and IGS-hybrid specimens from the coastal (Ayame) and the inland (Petessou) villages in Côte d’Ivoire. Left panel: results from genotyping of 18 Ancestry Informative Markers (AIMs, [21]) on chromosomal arms X, 2R, 3R, and 3L. Individuals are defined as admixed (adm) when at least 1 locus on chromosome- X or >1 out of 8 autosomal loci is not consistent with the other loci. Right panel: results from genotyping of 10 AIMs on chromosome-X. Individuals are defined as admixed (adm) when at least 1 locus is non consistent with the other loci. Italics = specimens identified discordantly by the IGS- and the multilocus approach.

		Multilocus ID			X- Multilocus ID			
Village	IGS-ID	CO	adm	GA	CO	adm	GA	Total
**Coastal**	CO	10	-	-	10			10
	GA/CO	*4*	4	-	4	4		8
	GA	*1*	*6*	4	1	2	8	11
	**Total**	**15**	**10**	**4**	**15**	**6**	**8**	**29**
**Inland**	CO	8	-	-	8			8
	GA/CO	-	-	*1*			1	1
	GA	*1*	*1*	8	1	1	8	10
	**Total**	**9**	**1**	**9**	**9**	**1**	**9**	**19**

## Data Availability

All data are available within the article and its Supplementary Material.

## Supplementary Materials

The following supporting information can be downloaded at: https://www.mdpi.com/article/10.3390/insects13121090/s1. Table S1: Information on Ancestry Informative SNPs genotyped and primer sequences used for the amplicon sequencing approach. Table S2: Genotyping results obtained for 24 Ancestry Informative SNPs for IGS-*An.gambiae*, IGS-*An.coluzzii* and IGS-hybrids from a coastal (Ayame) and an inland (Petessou) village Côte d’Ivoire. Table S3: Details on scientific publications reporting *An.gambiae* species composition in Côte d’Ivoire since 2010.
